# Determination of the Prevalence From Clinical Diagnosis of Sacroiliac Joint Dysfunction in Patients With Lumbar Disc Hernia and an Evaluation of the Effect of This Combination on Pain and Quality of Life

**DOI:** 10.1097/BRS.0000000000003309

**Published:** 2019-04-18

**Authors:** Hilal Telli, Berrin Hüner, Ömer Kuru

**Affiliations:** a Physiotherapy and Rehabilitation Department, Faculty of Health Sciences, European University of Lefke, Mersin, Turkey; b Physical Medicine and Rehabilitation Clinic, Ministry of Health Okmeydani Training and Research Hospital, Istanbul, Turkey.

**Keywords:** disc hernia, dysfunction, lumbar, sacroiliac joint

## Abstract

A prospective cross-sectional study.

**Objective.:**

To evaluate the prevalence of sacroiliac joint dysfunction in patients with lumbar disc hernia and examine the variations in clinical parameters cause by this combination.

**Summary of Background Data.:**

Although one of the many agents leading to lumbar pain is sacroiliac dysfunction, little progress has still been made to evaluate mechanical pain from sacroiliac joint dysfunction within the context of differential diagnosis of lumbar pain.

**Methods.:**

Two hundred thirty-four patients already diagnosed with lumbar disc hernia were included in the study. During the evaluation, sacroiliac joint dysfunction was investigated using specific tests, pain levels with a Visual Analog Scale, and the presence of neuropathic pain using Leeds Assessment of Neuropathic Symptoms and Signs Pain Scale. Other clinical assessments were performed using the Beck Depression Inventory, Health Assessment Questionnaire, and Tampa Kinesiophobia Scale.

**Results.:**

63.2% of patients were female and 36.8% were male. Mean age was 46.72 ± 11.14 years. The level of sacroiliac joint dysfunction was 33.3% in the research population. In terms of sex distribution, the proportion of women was higher in the group with sacroiliac joint dysfunction (*P* < 0.05). No significant difference was observed in pain intensity assessed using a Visual Pain Scale between the groups (*P* > 0.05), but the level of neuropathic pain was significantly higher in the group with dysfunction (*P* < 0.05). In the group with sacroiliac joint dysfunction, the presence of depression was significantly higher (*P* = 0.009), functional capacity was worse (*P* < 0.001), and the presence of kinesophobia was higher (*P* = 0.02).

**Conclusion.:**

Our study results will be useful in attracting the attention of clinicians away from the intervertebral disc to the sacroiliac joint in order to avoid unnecessary and aggressive treatments.

**Level of Evidence:** 2

## Abstract 2

The level of sacroiliac joint dysfunction was 33.3% in the study population. Accordingly to the surveys were used in this study, the neuropathic pain level, also the presence of kinesiophobia and depression were significantly higher in the group with sacroiliac joint dysfunction. Additionally, functional capacity was observed worse.

The point prevalence of lumbar pain, which affects approximately 50% to 80% of people in industrialized Western societies at certain periods of their lives and that is one of the main causes of workforce losses, medical costs, and disability, is between 15% and 30%.^1–3^

2% to 3% of all lumbar pains are those developing in association with lumbar disc herniation (LDH). Seventy percent of cases occur in the 30 to 50 age group, while 10% appear after 60. LDH in childhood is very rare.^4^

One of the many agents leading to lumbar pain is sacroiliac dysfunction, the prevalence of which, based on clinical assessments, is 15% to 30%.^5^ The sacroiliac joint (SIJ) was first described as a joint causing lumbar pain by Goldwaith and Osgood in 1905.^6^ In a study from 1909 involving SIJ dissection in 50 cadavers and seven cases, Albee described this as a genuine, mobile joint, and reported that lumbar pain and sciatica emerge when it is injured.^7,8^

The joint has its own particular biomechanical characteristics, anatomical variations, and diffuse neural innervation. The clinical presentation and pain patterns of sacroiliac joint dysfunction (SJD) are therefore still controversial.

The purpose of this study was to establish the prevalence of SJD in patients with LDH and to examine the variations in clinical parameters cause by this combination.

## MATERIALS AND METHODS

This cross-sectional study investigated patients aged 20 to 60 presenting to the Okmeydani Training and Research Hospital Physiotherapy and Rehabilitation Clinic, Turkey, due to lumbar-leg pain between January and April 2015. Two hundred thirty-four patients with diagnoses of LDH confirmed by clinical and radiological evaluations were enrolled in the study.

Patients aged 20 to 60, diagnosed with LDH, with cognitive levels sufficient to complete the assessment forms, and agreeing to participate were enrolled. Patients with lumbar pain, the etiology of which was suspected to be inflammatory in character, with structural vertebral deformity or fracture, with severe and progressive neurological deficit, with a history of severe psychiatric disease, with substance and/or alcohol dependence, with uncontrolled diabetes mellitus (DM), malignancy, spinal infection or a history of vertebral surgery, and pregnant subjects were excluded.

Demographic characteristics such as patients’ age, height, body weight (BW), body mass index (BMI), marital status, education level, and occupational status, and information concerning history of trauma, and the character, duration, and site of pain were investigated and noted on a case report form.

Before undergoing physical examination, all patients were evaluated in terms of pain threshold measurements using a Visual Analogue Scale (VAS), using the Leeds Assessment of Neuropathic Symptoms and Signs Pain Scale (LANSS) to the determine character of pain, with the Beck Depression Inventory (BDI) for psychological state, using the Health Assessment Questionnaire (HAQ) for quality of life and the Tampa Scale for Kinesiophobia for aversion to movement.

Lumbar spine flexion, extension, and right-left lateral flexion ranges of motion were determined using a universal goniometer and clinical tests were evaluated at physical examination.

In terms of clinical tests, Lasegue test, contralateral Lasegue, and the femoral nerve stretch test were used to determine LDH and six provocation tests (distraction, compression, Gaenslen, thigh thrust, sacral thrust, and Fleksiyon- Abduction – Eksternal Rotasyon (FABER) tests) for the SIJ. Based on the data from previous studies, patients with three or more positive provocation tests were diagnosed with SJD.^11–13^ In addition, all patients were asked about SIJ pain (pain when rising from a seated position, getting out of cars, and climbing stairs), and the data were recorded.

### Sample Size

A power analysis using the formula n = t2pq /d2 was performed to determine the sample size. Based on those data we concluded that a minimum of 174 and a maximum of 322 patients would be required to achieve significant results, and 234 patients were enrolled.

### Statistical Analysis

The study data were expressed as mean plus standard deviation (SD) for constant variables values and as number and percentage for categoric variables. The chi-square test was used to analyze categoric variables. Results were evaluated at a 95% confidence interval at a significance level of *P* < 0.05. The analysis was performed on SPSS (Statistical Package for the Social Sciences - SPSS Inc., Chicago, IL) for Windows 16.0 software.

The Strengthening the Reporting of Observational Studies in Epidemiology (STROBE) guideline has been implemented in this manuscript.

## RESULTS

Two hundred thirty-four patients, 148 females (63.2%) and 86 males (36.8%), with a clinical diagnosis of LDH and with disk protrusion and/or extrusion identified at magnetic resonance imaging (MRI) were included in the study. The presence of SJD was determined in 78 (33.3%) of the 234 patients using provocation tests. Dysfunction in the SIJ was determined on the right in 52.6% of patients and on the left in 47.4%. Patients were divided into groups, with or without SJD, and then compared.

### Demographic Characteristics

The ages of the 234 subjects in the study ranged between 20 and 60, the mean age being 46.72 ± 11.14. There was no statistically significant difference between the groups in terms of height, body weight, BMI, age, marital status, education, or employment (*P* > 0.05). A significant difference between the groups was observed only in terms of sex, the presence of SJD being higher in women (*P* = 0.005). Demographic data are shown in Table 1.

**TABLE 1:** Comparison of Demographic Data Between the Groups

	No.	SJD	No SJD	*P* Value
Age	234	46.17 ± 11.61	47.82 ± 10.12	0.28
Height, cm	234	166.29 ± 7.99	165.51 ± 7.48	0.47
Body weight, kg	234	75.79 ± 11.72	77.01 ± 11.84	0.45
BMI^*^, kg/m^2^	234	27.55 ± 4.64	28.15 ± 4.21	0.34
Sex				
Female	148	59 (75.6%)	89 (57.1%)	0.005
Male	86	19 (24.4%)	67 (42.9%)	
Education level				
Illiterate	27	11 (14.1%)	16 (10.3%)	0.63
Literate	2	1 (1.3%)	1 (0.6%)	
Primary school	99	35 (44.9%)	64 (41%)	
Middle school	49	35 (44.9%)	33 (21.2%)	
High school	36	11 (14.1%)	25 (16%)	
University	21	4 (5.1%)	17 (10.9%)	
Occupation				
Housewife	95	36 (46.2%)	59 (37.8%)	0.60
Sedentary	24	6 (7.7%)	18 (11.5%)	
Physically tiring work	70	21 (26.9%)	49 (31.4%)	
Retired	45	15 (19.2%)	30 (19.2%)	
Marital status				
Single	51	21 (26.9%)	30 (19.2%)	0.18
Married	183	57 (73.1%)	126 (80.8%)	

^*^BMI, body mass index.

### Pain Characteristics

Comparisons were performed between the groups in terms of location of pain, pain in the hip (SIJ), remaining standing for long periods, walking some distance and pain when rising from a seated position, getting out of a car or walking upstairs. Statistically, significant variation was determined in terms of the location of pain and prolonged standing (*P* < 0.05). No statistically significant variation was observed at a comparison of the location of pain and pain arising with prolonged standing (*P* > 0.05). The findings are shown in Table 2.

**TABLE 2:** Pain Characteristics

	No	SJD	No SJD	*P* Value
Pain on prolonged standing				
No	23 (9.8%)	5 (6.4%)	18 (11.5%)	0.25
Yes	211 (90.2%)	73 (93.6%)	138 (88.5%)	
Pain on walking some distance				
No	78 (33.3%)	12 (15.4%)	66 (42.3%)	<0.001
Yes	156 (66.7%)	66 (84.6%)	90 (57.7%)	
Pain on rising from a chair				
No	89 (38%)	14 (17.9%)	75 (48.1%)	<0.001
Yes	145 (62%)	64 (82.1%)	81 (51.9%)	
Pain on getting out of a car				
No	111 (47.4%)	19 (24.4%)	92 (59%)	<0.001
Yes	123 (52.6%)	59 (75.6%)	64 (41%)	
Pain on climbing stairs				
No	85 (36.3%)	15 (19.2%)	70 (44.9%)	<0.001
Yes	149 (63.7%)	63 (80.8%)	86 (55.1%)	

No statistically significance was determined between the groups in terms of duration of pain, VAS at rest, VAS during activity, and nocturnal VAS scores (*P* = 0.79, *P* = 0.96, *P* = 0.13, and *P* = 0.07, and *P* > 0.05, respectively).

### Imaging Findings

Although greater lumbosacral joint and SIJ degeneration were determined at radiological imaging in the SJD group, the differences were not statistically significant (*P* = 0.33 and *P* > 0.05, respectively).

Examination of lumbar MRI findings revealed hernial protrusion in 94.9% (n = 222) of patients and extrusion in 5.1% (n = 12), but no statistically significant difference was observed between the two groups (*P* = 0.75; *P* > 0.05). Examination of lumbar disk hernia levels revealed L5–S1 disk herniation in 53% of patients, L4–5 in 38.5%, L3–4 in 8.1%, and L2–3 in 0.4%. No significant variation was determined between the two groups (*P* = 0.70; *P* > 0.05).

### Examination Findings

Tests aimed at assessing LDH revealed no statistically significant difference between the groups in terms of Lasegue test, contralateral Lasegue, and the femoral nerve stretch test (*P* = 0.18, *P* = 0.13, and *P* = 1.00, *P* > 0.05, respectively). However, a statistically significant difference was observed in tenderness along the sciatic nerve (Valleix point) (*P* = 0.003, *P* < 0.05). No statistically significant difference was determined between the groups in terms of lumbar range of motion, hand-ground distance sensory deficit (root level), or decreased deep tendon reflexes in the patellar and Achilles tendons (*P* = 0.55, *P* = 0.76, *P* = 0.71, *P* = 0.33, and *P* = 1.00, *P* > 0.05, respectively).

No statistically significant difference was determined between the groups at evaluation of extremity length measurements (*P* = 0.72, *P* > 0.05), while comparison of SIJ provocation tests revealed significant variation in increasing pain with the Compression Test and the FABER, Gaenslen, Posterior Shear Test (POSH), and Sacral Thrust tests (*P* < 0.001).

### Questionnaires

Significant variation was observed between the groups in terms of the Beck Depression Scale, LANSS pain scale, HAQ, and Tampa kinesophobia scale (*P* < 0.05).

When BDI results were evaluated, the presence of moderate and severe depression was significantly higher in the SJD group, while in the non-SJD group, the non-depression group was significantly higher.

Patients were also evaluated using LANSS for neuropathic pain. The findings revealed that the presence of neuropathic pain was significantly higher in the SJD group.

The HAQ was used to evaluate the quality of life in our population and quality of life was significantly lower in SJD group.

Aversion to movement, evaluated using the Tampa Scale for Kinesophobia, was significantly higher in the group with SJD.

The findings are shown in Table 3.

**TABLE 3:** Questionnaires

	No	SJD	No SJD	*P* Value
Beck Depression Inventory				
No depression (<14)	168 (71.8%)	46 (59%)	122 (78.2%)	0.009
Moderate depression (14–24)	41 (17.5%)	20 (25.6%)	21 (13.5%)	
Severe depression (>24)	25 (10.7%)	12 (15.4%)	13 (8.3%)	
Total	234 (100%)	78 (100%)	156 (100%)	
LANSS^†^				
<12	170 (72.6%)	47 (60.3%)	123 (78.8%)	0.003
>12	64 (27.4%)	31 (39.7%)	33 (21.2%)	
HAQ^*^	234	0.87 ± 0.31	0.69 ± 0.38	<0.001
Tampa kinesophobia scale	234	25.18 ± 9.39	22.47 ± 7.37	0.02

^*^HAQ, Health Assessment Questionnaire.

^†^LANSS, Leeds Assessment of Neuropathic Symptoms and Sign.

## DISCUSSION

The purpose of this study was to determine the incidence of SJD in patients diagnosed with LDH through clinical examination and lumbar MRI presenting with lumbar-leg pain to the Okmeydani Training and Research Hospital PTR clinic and to assess the effect of this combination on clinical status, pain, and quality of life.

No significant difference was determined between the SJD and non-SJD groups in terms of age, BMI, marital status, or education levels. The incidence of SJD was higher among women in this study. Madani *et al*^9^ described female sex, a history of recurring lumbar pain and a heavy workload as significant risk factors for SJD. In that study, 55.9% of patients with SJD were female. It has been suggested that the greater incidence of SJD in female patients may be due to the effects on the SIJ of fertility, lifestyle, or low levels of exercise.^9^

Some studies have reported a decrease of at least 80% in VAS scores with local anesthesia injection guided by fluoroscopy, the gold standard technique for the diagnosis of SJD syndrome.^10^ Since SIJ blocks only provide information about pathologies of joint origin and SJD developing secondary to pathologies of structures around the joint can be overlooked, SIJ provocation and palpation tests also need to be used for diagnosis. The studies evaluating the validity and reliability of provocation tests in the diagnosis of sacroiliac joint dysfunction reported that three or more positive provocative tests, which include especially Ganslen, FABER, thigh thrust (POSH), compression and distraction tests, were highly sensitive and specific.^11–13^ However, all of these studies reported that should be used multiple tests for the diagnosis of SIJD and individual tests exhibited low reliability. Dreyfuss *et al*^14^ examined SI joint tests in a group consisting of asymptomatic individuals and observed symptoms of SIJD in 20% of asymptomatic patients. van der Wurff *et al*^15^ reported that the presence of three or more positive provocation tests in patients with pain deriving from the SI joint varied between 65% and 93%. Six provocation tests used in the diagnosis of SJD were performed on all patients in this study, and SJD was confirmed in the presence of three or more positive results.

The prevalence of SJD has been reported at 24% to 72.3% in previous studies.^9,17,20,21^ Madani *et al*^9^ investigated 202 patients aged 19 to 70, with LDH, determined at imaging and with physical findings of lumbosacral root irritation and reported SJD in 146 (72.3%) subjects. The authors reported that the estimated prevalence of SJD being significantly higher (72.3%) than that in previous studies^16–19^ might be explained in terms of the importance of non-joint factors that cannot be identified with SJD blocks.^9^

The prevalence of SJD in this study was 33.3%. SJD was located on the right in 52.6% of patients and on the left in 47.4%. Madani *et al*^9^ reported involvement on the left in 95.2% of patients with SJD, on both right and left in 2.1% and on the right in 2.7%. Slipman *et al*^20^ reported SJD on the right in 45% of cases, on the left in 35%, and bilateral in 20%.

Fortin *et al*^22^ reported the need to establish a pain chart in the diagnosis of SJD and showed on the basis of that chart that pain in patients with SJD was generally unilateral in the posterior superior iliac spine region. Pain diffusion map data are consistent with those of several previous clinical studies.^15,19,23–29^

In terms of pain distribution, van der Wurff *et al*^15^ reported that in SJD the patient directly indicated the painful joint. Weksler *et al*^21^ concluded that pain in 50 cases of lumbar pain and LDH diagnosed with SJD was distributed at 36% in the hip, inguinal in 26%, in the knee in 14%, in the calf in 14%, and in the foot in 10%.

Slipman *et al*^30^ reported hip pain in 94% of patients with SJD, lumbar pain in 72%, lower extremity pain in 50%, leg pain descending below the knee in 28%, and pain in the foot in 1%.

Analysis of pain location in the patients in our study population revealed that subjects most commonly presented with lumbar-leg pain, if no discrimination is made between right and left sides, and with lumbar and lumbar-left leg pain if right and left sides are differentiated. When the SJD and non-SJD groups were analyzed separately, greater lumbar-leg pain was observed in both groups than lumbar pain. However, when right and left side pain location differentiation was performed, right or left lumbar-leg pain was greater than lumbar pain in the SJD group, while in the non-SJD group lumber pain was greater than right or left lumbar-leg pain.

Evaluation of pain in the hip and gluteal region revealed no hip pain in 28.6% of patients, while in subjects with hip pain, levels of right and left side pain was similar, but slightly higher on the left. In the SJD group, the pain was present in 73.3% of patients, and pain in the left hip and the gluteal region was more prevalent than pain on the right. In the non-SJD group, the distributions of patients with no hip pain and of patients with pain in the right or left hip were approximately equal.

Evaluation of the BDI results in the patient population showed that subjects without depression constituted the majority in both groups. In the SJD group, the presence of moderate and severe depression was significantly higher, while in the non-SJD group, the non-depression group was significantly higher. These findings show that the prevalence of depression was higher in the SJD group and that such patients should be particularly evaluated in terms of depression.

Since our study evaluated patients with LDH, which directly concerns the neural stems, and considering the proximity of the SIJ to neural structures, patients were also evaluated using LANSS for neuropathic pain. The findings revealed neuropathic pain in 39.7% of the SJD group and in 21.2% of the non-SJD group. On the basis of these data, a significant difference was determined the prevalence of neuropathic pain was greater in the group with SJD. In addition, evaluation of sensitivity along the sciatic nerve (Valleix points) revealed 46.2% positivity in the SJD group and 26.3% positivity in the non-SJD group. On the basis of these findings, the presence of sensitivity along the sciatic nerve (Valleix points) was significantly higher in the group with SJD. In the literature, the presence of sensitivity and neuropathic pain along the sciatic nerve is thought to be associated with neighboring piriformis muscle spasm and sciatic nerve irritation secondary to SJD pathologies.^31^

Cheng and Ferrante^32^ compared patients diagnosed with lumbar radiculopathy and patients with SJD, assessing McGill pain scores, visual analog scores, and short form-36 quality of life scales. They concluded that there was no statistically significant difference between the patients with SJD and those with lumbar radiculopathy. The HAQ was used to evaluate the quality of life in our population and in agreement with the literature, statistically significant elevation was determined the group with LDH and SJD, indicating poor quality of life.

Aversion to movement, evaluated using the Tampa Scale for Kinesophobia, was significantly prevalent in the group with SJD, similarly to the prevalence of poor quality of life. Our scan of the literature revealed that the Tampa Scale for Kinesophobia and BDI had not previously been used in studies of patients with SJD. We think that in the light of the determination in this study of the fact that the presence of SJD accompanying LDH causes an increase in the prevalence of both kinesophobia and depression, these two conditions should be assessed separately both in daily practice and in future studies.

There are a number of significant limitations to this study. The inclusion of patients with lumbar pain secondary to LDH may have led to the relation between the prevalence of SJD and other clinical entities being overlooked. In addition, fluoroscopy-guided local anesthesia injection into the SIJ, one of the criteria set out in the diagnosis of SJD by the International Association for the Study of Pain in 1994, was not performed. However, we think that the appropriate patient number in the study population, an upper age limit of 60 being imposed, rheumatological diseases being regarded as an exclusion criterion and patient examinations and all assessments being performed by a single physician being under equivalent conditions for all patients increase the reliability and validity of the data.

In conclusion, SJD is a pathology accompanying disc degeneration at a level of approximately one in three in patients with LDH, and this condition should be considered when planning a conservative treatment protocol for patients with lumbar or lumbar-leg pain. SIJ involvement should be determined using SIJ provocation tests in patients with LDH, particularly when conservative treatment is unsuccessful. In this way, the attention of physicians and surgeons can be diverted from the intervertebral disk to the SIJ for the avoidance of unnecessary lumbar region surgery based on pain indications.

**Key Points**

- One of the many agents leading to lumbar pain is sacroiliac dysfunction, the prevalence of which, based on clinical assessments, is 15% to 30%.
- The level of sacroiliac joint dysfunction was 33.3% in our research population. In terms of sex distribution, the proportion of women was higher in the group with sacroiliac joint dysfunction.
- The level of neuropathic pain, the presence of depression and kinesophobia were significantly higher in the group with dysfunction.
- In addition, functional capacity was worse in the group with sacroiliac joint dysfunction.
